# Implant-supported fixed prostheses with cantilever: a systematic review and meta-analysis

**DOI:** 10.1186/s40729-024-00573-8

**Published:** 2024-11-21

**Authors:** Yusuke Kondo, Kiyoshi Sakai, Hajime Minakuchi, Takuya Horimai, Takuo Kuboki, JSOI Clinical Guideline Working Group collaborators, Ikiru Atsuta, Akira Hasuike, Kazuki Takaoka, Norimasa Tanabe, Kensuke Yamauchi, Hideki Aita, Kentaro Akiyama, Junichi Furuya, Kazuhiko Hiroyasu, Masamitsu Oshima, Yohei Sato, Takashi Sawase, Yoichi Yamada, Yasunori Ayukawa, Yoko Kurosaki, Takuya Mino, Takashi Sakamoto, Hisatomo Kondo, Takashi Miyazaki, Ryuji Hosokawa

**Affiliations:** 1 Clinical Guideline Task-Force Members (2018-), Japanese Society of Oral Implantology (JSOI), Tokyo, Japan; 2 Committee Members for Research Planning and Promotion (2020-2021), JSOI, Tokyo, Japan; 3 Committee Members for Research Planning and Promotion (2022-2023), JSOI, Tokyo, Japan; 4 Committee Chairs for Scientific Research on Oral Implantology: Takashi Sakamoto (2020-2021), Hisatomo Kondo (2022-), JSOI, Tokyo, Japan; 5 Contributing Presidents of JSOI: Takashi Miyazaki (2018-2021), Ryuji Hosokawa (2022-), Tokyo, Japan; 6 Division of Oral Reconstruction and Rehabilitation, Kyushu Dental University, Kitakyushu, Japan; 7 Department of Oral and Maxillofacial Surgery, Nagoya University Hospital, Nagoya, Japan; 8 Department of Oral Rehabilitation and Implantology, Okayama University Hospital, Okayama, Japan; 9 The Library, School of Dentistry, Nihon University, Tokyo, Japan; 10 Department of Oral Rehabilitation and Regenerative Medicine, Okayama University Faculty of Medicine Dentistry and Pharmaceutical Sciences, Okayama, Japan; 11 Section of Fixed Prosthodontics, Division of Oral Rehabilitation, Faculty of Dental Science, Kyushu University, Fukuoka, Japan; 12 Department of Periodontology, Nihon University School of Dentistry, Tokyo, Japan; 13 Department of Oral and Maxillofacial Surgery, Shiga University of Medical Science, Otsu, Japan; 14 Department of Prosthodontics and Oral Implantology, School of Dentistry, Iwate Medical University, Morioka, Japan; 15 Division of Oral and Maxillofacial Reconstructive Surgery, Department of Disease Management Dentistry, Tohoku University Graduate School of Dentistry, Sendai, Japan; 16 Division of Geriatric Dentistry, Department of Human Biology and Pathophysiology, School of Dentistry, Health Sciences University of Hokkaido, Tobetsu, Japan; 17 Department of Oral Function Management, Graduate School of Dentistry, Showa University, Tokyo, Japan; 18 Oral Implant Care Unit Niigata Hospital, The Nippon Dental University, Niigata, Japan; 19 Department of Stomatognathic Function and Occlusal Reconstruction, Institute of Biomedical Sciences, Clinical Dentistry, Tokushima University Graduate School, Tokushima, Japan; 20 Department of Oral Rehabilitation and Prosthodontics, ,Tsurumi University School of Dental Medicine, Yokohama, Japan; 21 Department of Applied Prosthodontics, Graduate School of Biomedical Sciences, Nagasaki University, Nagasaki, Japan; 22 Department of Oral and Maxillofacial Surgery, Gifu University Graduate School of Medicine, Gifu, Japan; 23 Section of Implant and Rehabilitative Dentistry, Division of Oral Rehabilitation, Faculty of Dental Science, Kyushu University, Fukuoka, Japan; 24 Department of Removable Prosthodontics and Occlusion, Osaka Dental University, Osaka, Japan; 25 Clinical Academy of Oral Implantology, Osaka, Japan; 26 Department of Fixed Prosthodontics and Oral Implantology, School of Dentistry, Aichi Gakuin University, Nagoya, Japan; 27 Showa University, Tokyo, Japan

**Keywords:** Cantilever, Fixed prostheses, Implants, Partial edentulism, Systematic review

## Abstract

**Purpose:**

This systematic review (SR) aimed to investigate whether the presence of a cantilever affects the results of implant treatment for partial edentulism, including an analysis of the anterior and posterior regions of the dental arches.

**Methods:**

An electronic search was performed, and original articles published between 1995 and November 2023 were included. The outcomes were the implant survival rate, patient satisfaction, occurrence of mechanical complications, and marginal bone loss around the implants. Two SR members independently examined the validity of the studies, extracted evidence from the included studies, and performed risk of bias assessment, comprehensive evidence evaluation, and meta-analysis.

**Results:**

Nine studies met our inclusion criteria. Implant survival rate tended to be lower in the cantilever group, and marginal bone loss tended to be higher in the cantilever group; however, there was no significant difference. There was no significant difference in patient satisfaction based on the presence or absence of a cantilever. Moreover, the incidence of mechanical complications was significantly higher in the cantilever group. According to the analysis of anterior and posterior regions, implant survival rate tended to be lower in the cantilever group of the posterior region, and marginal bone loss around the implants tended to be higher in the cantilever group of the anterior region.

**Conclusion:**

Implant-supported fixed prostheses with cantilevers did not negatively affect implant survival rate, marginal bone loss, or patient satisfaction. However, the incidence of mechanical complications significantly increased in the cantilever group.

## Background

Although restoring occlusal function with fixed prostheses for multiple missing teeth, supporting both ends of the edentulous area with implants is effective in achieving mechanical stability. When implant placement in the ideal position is difficult owing to insufficient bone volume or anatomical constraints, bone augmentation is performed. However, bone augmentation is sometimes associated with surgical stress and a prolonged treatment duration [1]. These problems can be avoided using a prosthesis with a cantilever. By applying prostheses with a cantilever, risks such as damage to the nerves and adjacent teeth can be avoided and the treatment period can be shortened [2]. As the cantilever structure has a large distance between the fulcrum and point of action, which increases the moment of occlusal force applied to the implants, it cannot be considered mechanically ideal [3, 4], consequently, it can be difficult to decide whether or not to use cantilevers in clinical practice.

To date, two systematic reviews (SRs) regarding the prognosis of implant treatment with cantilevers, including several outcomes such as implant survival rate, marginal bone loss around implants, and the occurrence of mechanical complications, have been published. These SRs suggest that the presence of a cantilever may not have a negative effect on implant treatment, except for mechanical complications [5, 6]. However, the number of studies included in previous SRs was relatively small. Over seven years have passed since the last published literature search for existing SRs, and there is a high likelihood that more studies have been published; therefore, this information needs to be updated. Furthermore, although conditions, such as the occlusal force applied to the implants differ between the anterior and posterior regions of the dental arches, there has been no analysis for each region. Hence, the purpose of this SR was to update information regarding the prognosis of implant-supported fixed prostheses with cantilevers for partial edentulism, including an analysis of the anterior and posterior regions, in order to provide guidance for clinical treatment decisions.

## Methods

### Clinical question and participant/patient, intervention, comparison, and outcome approach

This SR was conducted in accordance with the guidelines recommended by the Preferred Reporting Items for Systematic Reviews and Meta-Analysis (PRISMA 2020) statement [7]. The review protocol was structured according to the Medical Information Network Distribution Service Practice Guideline Development Manual 2020 [8]. The scope of this SR was registered in PROSPERO (PROSPERO No. CRD42024559399) [9].

This SR examined the clinical prognoses of cantilever-type superstructures in patients with implant-supported fixed prostheses. The following review questions were formulated using participant/patient, intervention, comparison, and outcome (PICO) approach. The clinical question (CQ) in this SR was: “Are cantilevered implant-supported fixed prostheses in dental implant treatment effective in cases of partial edentulism compared to conventional fixed prostheses?” The PICO was set as follows:

P: Patient with multiple teeth loss.

I: Installation of cantilevered implant-supported fixed prosthesis.

C: Installation of non-cantilevered implant-supported fixed prosthesis.

O: Implant survival rate, patient satisfaction, mechanical complications, and marginal bone loss around implants.

### Search strategy

To systematically and reliably collect relevant studies, we conducted an electronic search of databases (PubMed, Cochrane Central Register of Controlled Trials [CENTRAL], and Ichushi-Web) targeting original articles written in English or Japanese published between 1995 and November 30, 2023. The search terms included keywords (Medical Subject Headings) and free-text terms, and search expressions were constructed using Boolean operators (OR and AND). The construction of this search formula was commissioned by a librarian independent of the TF clinical practice guidelines to ensure fairness.

**Fig. 1:**
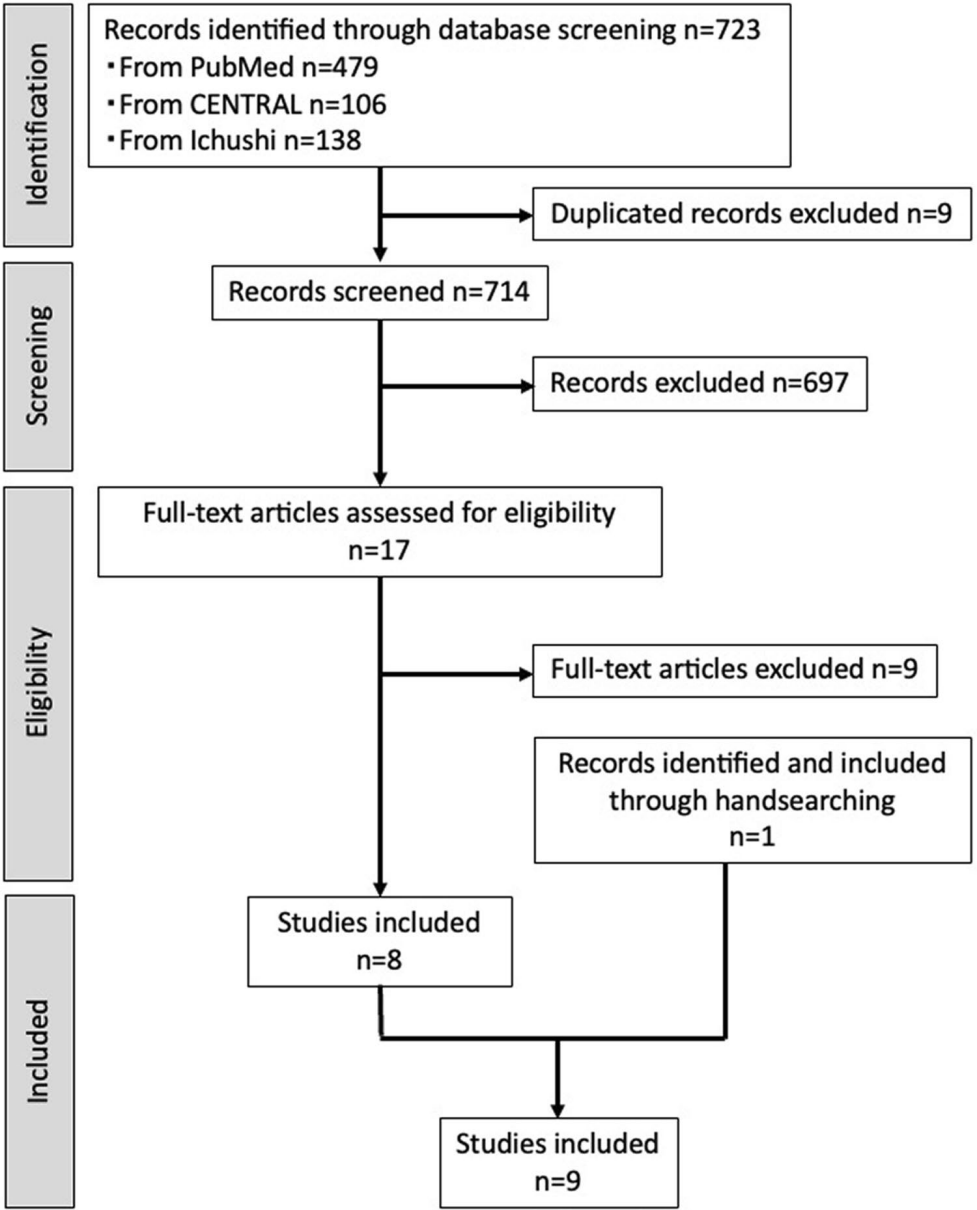
Study selection flowchart

The search was performed using the following terms: A, implant-supported fixed prostheses; B, cantilever-related terms; C, trouble incidence/marginal bone loss around implants; and D, prognosis, narrowed down by language and year. A similar search was performed in both CENTRAL and Ichushi-Web databases. Figure 1A shows the search formulas used in PubMed and CENTRAL, and Fig. 1B shows the search formula used in Ichushi-Web. The PubMed, CENTRAL, and Ichushi-Web searches were performed on November 30, 2023, February 6, 2024, and February 8, 2024, respectively. We also conducted manual searches.

### Inclusion and exclusion criteria

The inclusion criteria for selecting documents from which to extract evidence were randomized controlled trials (RCTs), cohort studies, and case–control studies written in English or Japanese. The exclusion criteria were as follows: ineligible target subjects; ineligible evaluation method; ineligible research design; main text in languages other than English or Japanese; and narrative reviews, letters, and review articles. If an applicant was judged ineligible for reasons other than those listed above, the reason for exclusion was clearly stated. If duplication was possible, the latest publication was included.

### Screening method

Two SR members independently examined the validity of each document on the CQ in two stages. The first stage was screening based on study titles and abstracts. In the second stage, the entire literature was collected and two SR members independently examined the validity of the study according to the aforementioned criteria. Subsequently, the results for each SR member were compared. In case of disagreement between SR members, discussions were held until a consensus was reached, and evaluation was performed after the agreement was reached.

### Data extraction

Data extraction was performed independently by two SR members using a standardized form that included information on authors, title, year of publication, journal, study design, level of evidence, number of subjects, observation period, and outcomes.

### Risk of bias assessment

The quality of each study was assessed simultaneously using a data extraction process. The quality of the obtained evidence was assessed by evaluating five factors: risk of bias, indirectness, imprecision, inconsistency, and publication bias [10–13]. These assessments were conducted by two SR members who extracted evidence using tools from The Cochrane Collaboration [14].

### Meta-analysis

Using Review Manager 5.4 (The Cochrane Collaboration), a meta-analysis was performed on three outcomes: implant survival rate, mechanical complications, and marginal bone loss around the implants. The Mantel–Haenszel test was used for statistical analysis, and significance was set at *p* < 0.05. Random effects model was used to account for variability between studies. Separate analyses were performed for the anterior and posterior regions regarding the implant survival rate and marginal bone loss around the implants. In addition, funnel plots were created to determine whether there was the likely possibility of publication bias.

## Results

### General outcomes

The studies extracted from the databases using the search formula included 479 studies in PubMed, 106 in CENTRAL, and 138 in Ichushi-Web. Nine articles were duplicated, resulting in a total of 714 studies. After screening, 697 articles were excluded, and after examining the validity of the entire literature, nine articles were excluded, resulting in eight articles. Furthermore, one article was included in the manual search; consequently, nine studies were included (Fig. 1): one RCT and eight cohort studies, and the details of each study are shown in Table 1. The results of each study are summarized in Table 2. The risk of bias assessment and body of evidence for each outcome are shown in Tables 3 and 4, respectively. The results for each outcome and validity of the evidence are described below.

**Table 1:** Database search strategies

A	#1	"dental prosthesis, implant supported"[MeSH Major Topic] OR "dental prosthesis, implant supported/adverse effects"[MeSH Terms]
	#2	implant*[TIAB] AND support*[TIAB] AND (prosthes*[TIAB] OR denture*[TIAB])
	#3	#1 OR #2
B	#4	extension[TIAB] OR pontic[TIAB] OR cantilever*[TIAB] OR crowns[TIAB] OR "porcelain fracture"[TIAB]
C	#5	adverse effects [Subheading] OR Alveolar Bone Loss[MH] OR Dental Restoration Failure[MH]
	#6	"marginal bone"[TIAB] OR "bone loss"[TIAB] OR failure*[TIAB] OR complication*[TIAB]
	#7	#5 OR #6
D	#8	Retrospective Studies[MH] OR Prospective Studies[MH] OR Treatment Outcome[MH] OR Survival Analysis[MH]
	#9	(retrospective*[TIAB] AND stud*[TIAB]) OR (prospective*[TIAB] AND stud*[TIAB])
	#10	#8 OR #9
	#11	#3 AND #4 AND #7AND #10
	#12	1995/1/1:2023/11/30[Date—Entry] AND ("English"[Language] OR "Japanese"[Language])
	#13	#11 AND #12
	#1	Dental implant/MTH or Dental implant material/MTH or peri-implantitis/MTH or Implant-supported prosthesis/MTH or Implant/TI
	#2	Cantilever/TA or pontic/TA or Extended bridge/TA or extension/TA or extention/TA or pontic/TA or cantilever/TA
	#3	#1 and #2
	#4	(DT = 1995:2023 and PT = Exclude meeting abstract)
	#5	#5 and #6

**Table 2:** Data collected from included studies

Study	Study design	Age (year)	Sex	Sample size (Number of patients)	Sample size (Number of prosthesis)	Sample size (Number of implants)	Implant posision	Implant characteritics	Follow-up
Wennstrom 2004	Cohort study	Cantilever gourp 57 ± 10.3 Control group 62 ± 8.5	17 Male and 30 Female	Cantilever gourp 24 Control group 23	Cantilever gourp 24 Control group 26	unknown	unknown	Astra Tech	5y
Halg 2008	Cohort study	24–83	21 Male and 33 Female	Cantilever gourp 27 Control group 27	Cantilever gourp 27 Control group 27	Cantilever gourp 46 Control group 32	Posterior region	Straumann	3–12.7y
Tymstra 2011	Cohort study	Cantilever gourp 20–43 Control group 18–49	6 Male and 4 Female	Cantilever gourp 5 Control group 5	Cantilever gourp 5 Control group 5	Cantilever gourp 5 Control group 10	Anterior region	Nobel biocare	1y
Kim 2014	Cohort study	Cantilever gourp 61.9 ± 10.2 Control group 62.37 ± 11.4	88 Male and 118 Female	Cantilever gourp 107 Control group 99	Cantilever gourp 123 Control group 144	Cantilever gourp 132 Control group 203	Anterior and posterior region	MIS, Neoss, 3i, Nobel Biocare	Cantilever group 51 ± 33 m Control group 49 ± 27 m
Mumcu 2014	Cohort study	55.0 ± 12.2	15 Male and 21 Female	Cantilever gourp 16 Control group 20	Cantilever gourp 16 Control group 20	unknown	Anterior and posterior region	Astra Tech, Straumann, Zimmer Dental, Biohorizons	3y
Taha 2020	Randomized controlled trial	40 (30–50)	6 Male and 8 Female	Cantilever gourp 7 Control group 7	Cantilever gourp 7 Control group 7	Cantilever gourp 14 Control group 14	Anterior region	Tixos implant	2y
Roccuzo 2020	Cohort study	Cantilever gourp 26 ± 3 Control group 27 ± 4	7 Male and 16 Female	Cantilever gourp 16 Control group 7	Cantilever gourp 16 Control group 7	Cantilever gourp 16 Control group 14	Anterior region	Astra Tech, Straumann	Cantilever group 33.3 ± 19.3 m Control group 47.6 ± 36.3 m
Derici 2021	Cohort study	57.9 ± 8.3	22 Male and 30 Female	Cantilever gourp 22 Control group 30	Cantilever gourp 22 Control group 30	Cantilever gourp 22 Control group 60	Posterior region	unknown	2y
Horsch 2022	Cohort study	Cantilever gourp 60.47 ± 9.25 Control group 62.85 ± 10.72	130 Male and 159 Female	Cantilever gourp 48 Control group 241	Cantilever gourp 48 Control group 241	Cantilever gourp 75 Control group 300	Anterior and posterior region	unknown	Cantilever group 3.56y Control group 7.25y

**Table 3:** Summary of risk of bias assessment in included studies

(A) Invtervention study											
Outcome		Survival of implant-retained fixed prostheses									
		Riak of bias									
		Selection bias		Performance bias	Detection bias	Attrition bias		Other bias			
Study	Study design	Random sequence generation	Allocation concealment	Blinding of participants and personnel	Blinding of outcome assessment	ITT	Incomplete outcome data	Selective reporting	stopped early trial for benefits	Other source of bias	Summary
Taha 2020	RCT	0	0	−1	0	0	0	0	0	0	0

(B) Observation studies								
Outcome		Survival of implant-retained fixed prostheses						
		Riak of bias						
		Selection bias	Performance bias	Detection bias	Attrition bias	Other bias		
Study	Study design	Random sequence generation	Allocation concealment	Blinding of participants and personnel	Blinding of outcome assessment	ITT	Incomplete outcome data	Selective reporting
Wennstrom 2004	Cohort study	−2	−1	0	−1	−1	0	−1
Halg 2008	Cohort study	−2	−1	0	−1	−1	0	−1
Tymstra 2011	Cohort study	−1	0	0	−1	−1	0	−1
Kim 2014	Cohort study	−2	−1	0	−1	−1	0	−1
Mumcu 2011	Cohort study	−2	−1	0	−1	−1	0	−1
Roccuzzo 2020	Cohort study	−1	−1	0	−1	−1	0	−1
Derici 2021	Cohort study	−1	−1	0	−1	−1	0	−1
Horsch 2022	Cohort study	−2	−1	0	−2	−1	0	−2

**Table 4:** Assessment of body of evidence

Outcome	Study design/number of studies	Risk of bias	Inconsistency	Imprecision	Indirectness	Other bias (publication bias)	Certaincy of evidence
Survival of implant-retained fixed prostheses	RCT/1 Cohort study/8	−1	0	−1	0	0	Very low (D)
Patients′ satisfaction	Cohort study/1	−1	N/A	−2	0	0	Very low (D)
Mechanical complication	Cohort study/3	−1	0	−1	0	−1	Very low (D)
Marginal bone loss	RCT/1 Cohort study/6	−1	−1	−1	0	−1	Very low (D)

### Implant survival rate

**Fig. 2:**
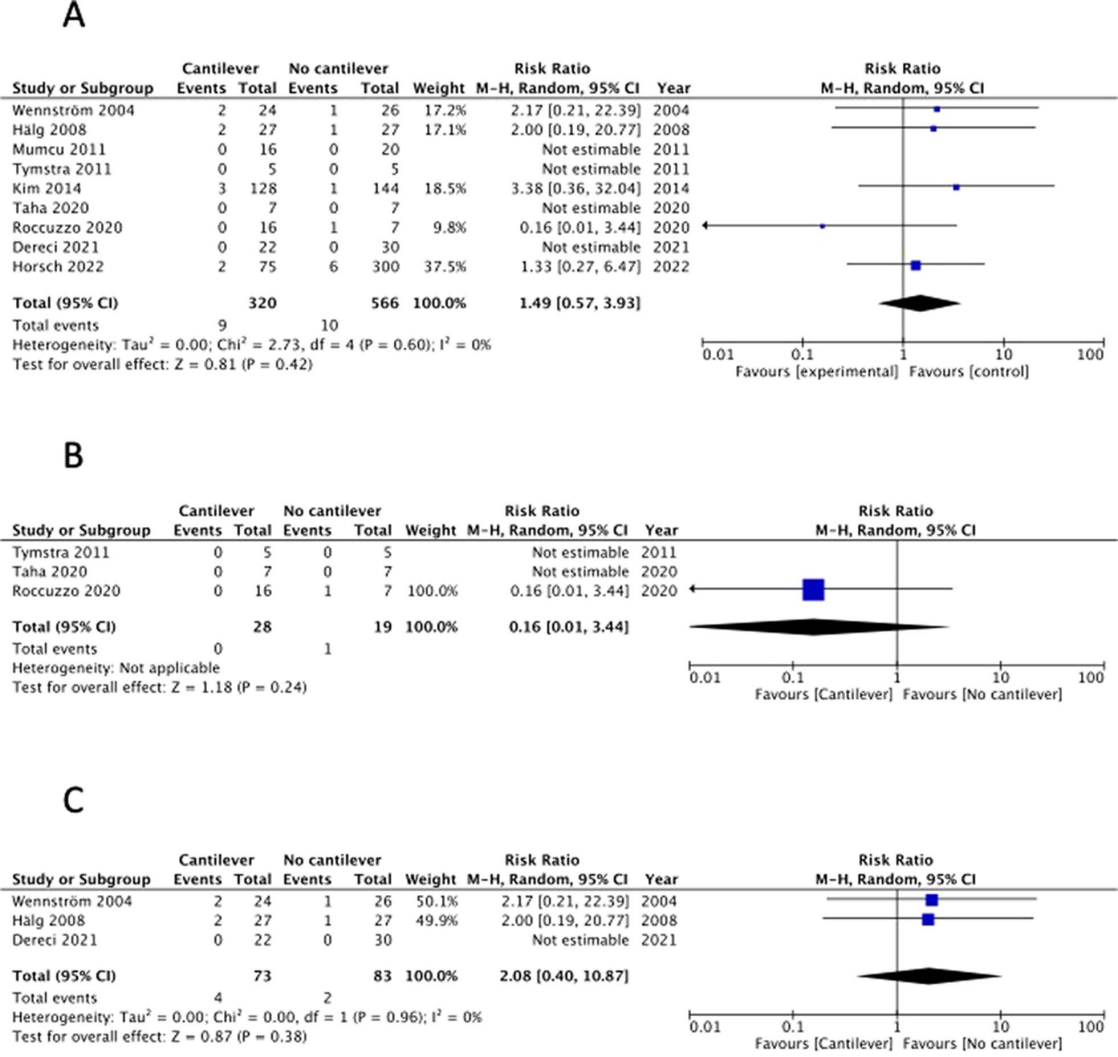
Forest-plot outcomes: survival of implant-retained fixed prostheses. **A** Overall analysis. **B** Anterior region analysis. **C** Posterior region analysis

There were 9 studies that included implant survival rate as an outcome [15–23]. Although the RCT reported by Taha et al. was not blinded, other biases were minimized; therefore, the risk of bias was judged to be low [19] (Table 3A). The remaining were cohort studies that did not undergo randomization and were judged to have a large selection bias (Table 3B). Moreover, detection bias was evaluated as low, and the overall risk of bias was evaluated as − 1. In addition, the inconsistency was evaluated as 0 because I^2^ = 0% (Fig. 2A). Further, owning to the small sample size, imprecision was evaluated as -1. The implant survival rate was consistent with the CQ as an outcome, and indirectness was evaluated as 0. Furthermore, the funnel plot for implant survival rate was symmetric, therefore publication bias was judged as 0 (Fig. 5A). When these were combined, the evidence was judged very low (Table 4).

### Patients’ satisfaction

Only one cohort study included patient satisfaction as an outcome [18]. As no randomization was performed, the selection bias was judged to be large. Additionally, other biases were also assessed as − 1, consequently the risk of bias was assessed as − 1 (Table 3B). Because only one study was included, inconsistency could not be rated. Imprecision was rated -2 because of the small sample size. Patient satisfaction was consistent with the CQ; therefore, indirectness was evaluated as 0. Based on above, evidence was judged very low (Table 4). The included studies showed that patient satisfaction was similar regardless of the presence of a cantilever.

### Mechanical complications

**Fig. 3:**
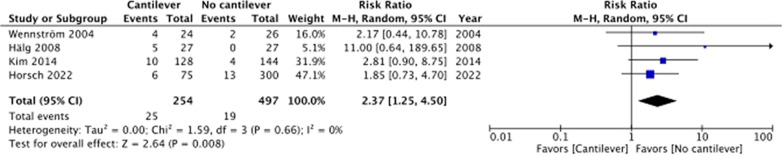
Forest-plot outcomes: mechanical complication rate of implants or implant-retained fixed prostheses

Four studies included the occurrence of mechanical complications as an outcome [15, 16, 21, 22]. Because all studies were cohort studies and no randomization was performed, the selection bias was judged to be large. Most of other biases were rated as moderate (Table 3B); therefore, the overall risk of bias was assessed as − 1 (Table 4). Inconsistency was rated 0 because I^2^ = 0% (Fig. 3). Owning to the small sample size, imprecision was rated as -1. Because the evaluated complications, such as loosening or fracture of screws and failure of prostheses, were consistent with the CQ, the indirectness was evaluated as 0. The funnel plot for mechanical complications was somewhat asymmetric, therefore publication bias was judged as − 1 (Fig. 5B). All the included studies reported that mechanical complications were more common in cantilevered implant treatments. However, the risk of bias was high, and the sample size was small; therefore, the evidence was judged to be very low (Table 4). The results of the meta-analysis showed that mechanical complications significantly increased in the cantilever group (Fig. 3). Because the number of studies that included mechanical complications as an outcome was small, a separate analysis by treatment region was not performed.

### Marginal bone loss around implants

**Fig. 4:**
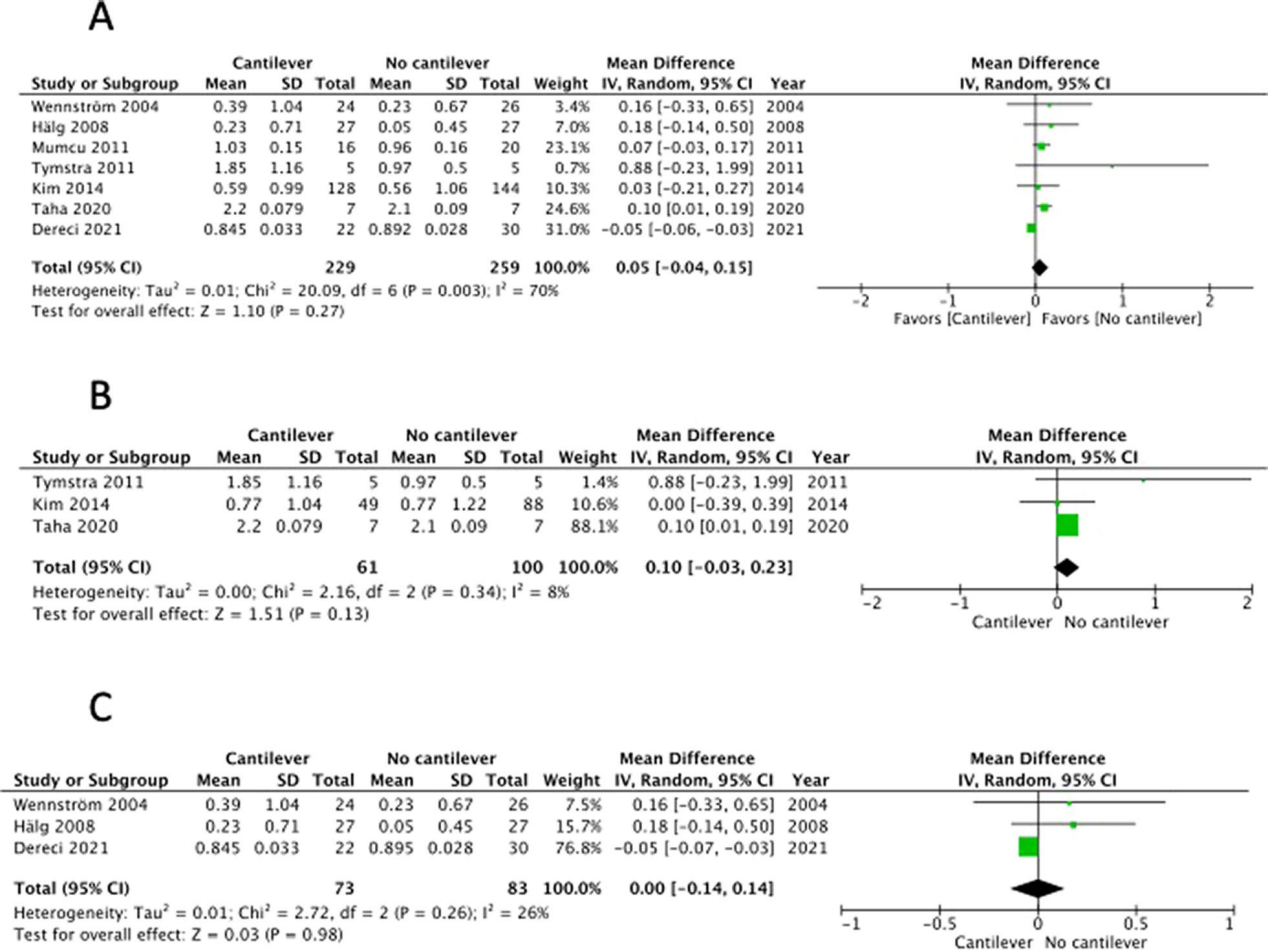
Forest-plot outcomes: marginal bone loss around implants. **A** Overall analysis. **B** Anterior region analysis. **C** Posterior region analysis

**Fig. 5:**
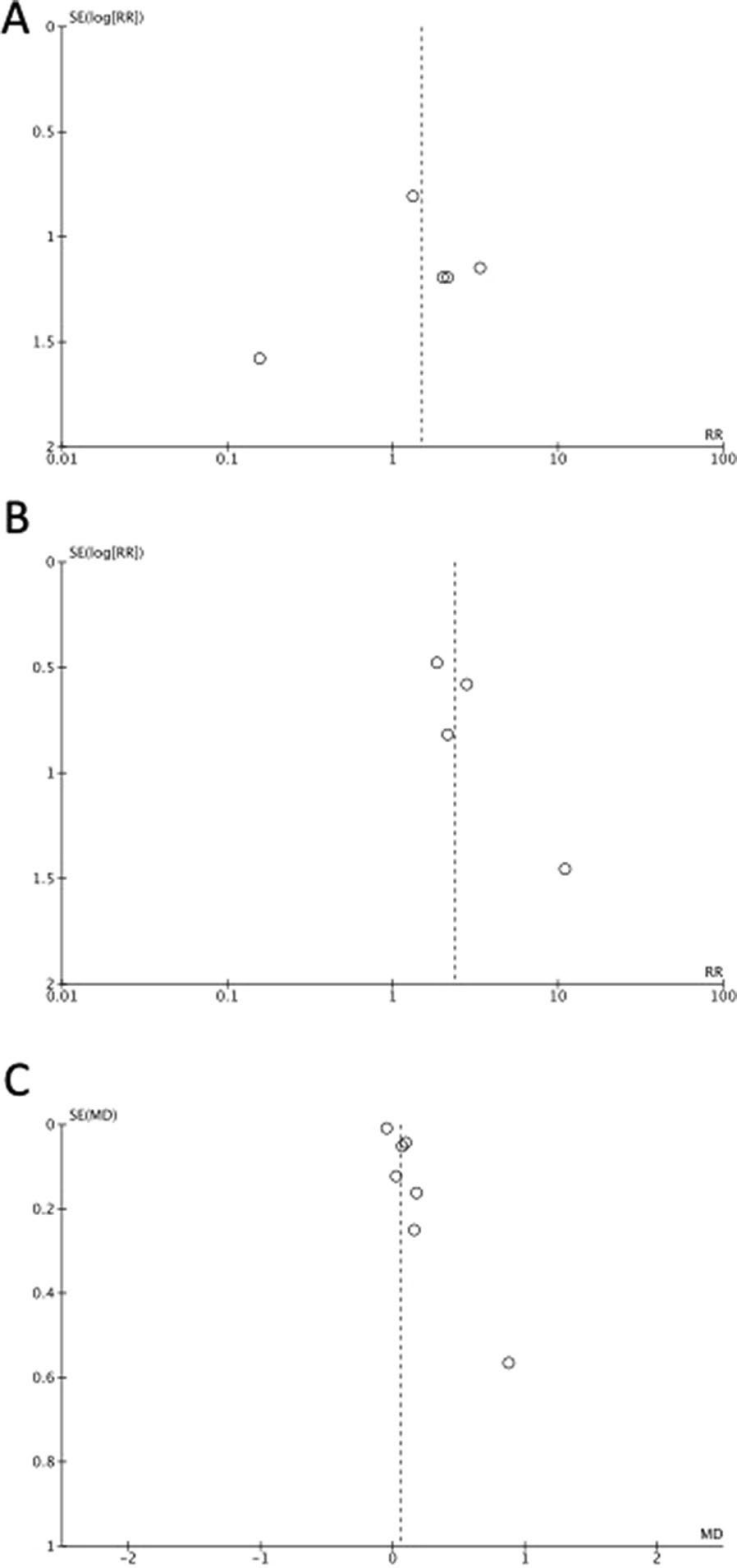
Funnel plots of **A** survival of implant-retained fixed prostheses; **B** mechanical complication rate of implants or implant-retained fixed prostheses; and **C** marginal bone loss around implants. SE (log[RR]): Standard error of log risk ratio, RR: risk ratio, SE (MD): Standard error of mean difference, MD: mean difference

Seven studies included marginal bone loss as an outcome, including one RCT [19] and six cohort studies [15–18, 20, 21]. One RCT [19] was not blinded, and the risk of bias was low (Table 3A). Further, six cohort studies were randomized, and selection bias was judged to be large, and the risk of bias was assessed as − 1. Inconsistency was rated as − 1 because I^2^ = 70% (Fig. 4A), imprecision was rated as − 1 because the sample size was small, and indirectness was rated as 0 because the evaluation of marginal bone loss was consistent with this CQ. The funnel plot for marginal bone loss was somewhat asymmetric, therefore publication bias was judged as − 1 (Fig. 5C). Based on the above, the evidence was judged as very low (Table 4). One RCT [19] reported that the marginal bone loss on the cantilever side of implants with cantilevers was significantly greater than that of implants without cantilevers. Five cohort studies [15–18, 21] reported no significant differences in marginal bone loss between implants with and without cantilevers. Furthermore, one cohort study [20] reported that the amount of bone resorption was significantly greater with cantilevered implants depending on the elapsed time; however, the difference was small, and there was no significant difference depending on the elapsed time. The risk of bias was high, and the number of studies was small; therefore, the evidence was judged to be very low (Table 4). Considering that this depends on the region, one RCT [19] focused on implant treatment of the anterior teeth. In this study, two implants were placed in the anterior region, and a bridge, with or without a cantilever, was attached as a prosthesis. Marginal bone loss was greater in implants located close to the cantilever than in other types of implants. Three cohort studies [18, 19, 21] included results from the anterior tooth region. In these studies, although there was no significant difference in marginal bone loss depending on the presence or absence of cantilevers, the marginal bone loss in the cantilever group tended to be slightly larger. However, results in the posterior region have been reported in three cohort studies [15–17], all of which reported no significant differences in marginal bone loss depending on the presence or absence of cantilevers. The results of the meta-analysis indicated that marginal bone loss tended to be greater in the cantilever group (Fig. 4A). When analyzed separately for the anterior and posterior regions, the marginal bone loss tended to be greater in the cantilever group in the anterior region (Fig. 4B); however, it was similar between both groups in the posterior region (Fig. 4C).

## Discussion

In this SR, we investigated whether the presence of a cantilever affects the results of implant treatment for partial edentulism, including an analysis of the anterior and posterior regions of the dental arches. Further, we comprehensively searched for studies on the prognosis of implant treatment using cantilevered implant-supported fixed prostheses and selected eligible studies. Consequently, nine studies met the inclusion criteria. Of these, eight were cohort studies and only one was an RCT. Nine articles [15–23] included implant survival rate as an outcome. Most showed a tendency for the implant survival rate to be slightly lower in the cantilever group or comparable between the cantilever and control groups. Therefore, the consistency of the results between studies was assessed to be high. However, the number of placed implants differed among the studies: two studies placed one implant [18, 23], two studies placed two implants [17, 19], and the number of placed implants was unknown in the other five studies [15, 16, 20–22]. Furthermore, although implant length and diameter influenced the implant survival rate [24, 25], these factors were not standardized among the included studies.

Although patient satisfaction is clinically critical, only one study included it as an outcome [18]. The study concluded that satisfaction levels were the same regardless of the presence or absence of a cantilever. However, in this study, only the morphology and color of the crown and gingiva were evaluated as outcomes of patient satisfaction, and there was a lack of evaluation of items such as cleanability that may be degraded by the presence of cantilevers. In the long term, mechanical complications, which increase in the presence of a cantilever, could reduce patient satisfaction. Furthermore, the presence of a cantilever often reduces surgical invasion, treatment costs, and treatment duration, which may lead to increased patient satisfaction. Further studies that include more patient satisfaction outcomes are required.

With regard to the occurrence of mechanical complications as an outcome, all four studies demonstrated that the presence of a cantilever increased mechanical complications. Because the cantilever structure is not mechanically superior, it is easy to imagine that mechanical complications can increase. Furthermore, the prosthesis material has a significant effect on the occurrence of mechanical complications. Among the studies included in this SR, one study used porcelain-fused-to-metal crowns as a prosthesis, whereas the other three studies did not mention the material of the prostheses. Future studies using prostheses made of other materials, such as monolithic zirconia or all-ceramic crowns, may lead to new findings. Furthermore, the prosthesis fixation method can affect the occurrence of mechanical complications [26]. Of the included studies, one used screw fixation [15] and the type of fixation was unclear in the other three studies [16, 21, 22]. Moreover, although the use of screw-retaining abutments could affect the frequency of mechanical complications, such as implant fractures, only one study mentioned the use of screw-retaining abutments [15].

Regarding the amount of marginal bone loss around the implants, the type of implant-abutment connection is a factor that influences marginal bone loss, and systems with conical connections have been reported to have smaller marginal bone loss [27]. However, only one study [15] clearly indicated the implant-abutment connection; in all other studies, the implant-abutment connection was unclear, or multiple connections were mixed within the study. In addition, in the case of screw fixation, the presence of a screw-retaining abutment affects marginal bone loss [28]; however, among the studies included in this SR, only one study specified the use of a screw-retaining abutment [15]; in other studies, it was unclear whether a screw-retaining abutment was used. Among the factors that can cause marginal bone loss, mechanical factors and cleanability have been proposed as factors related to the cantilever. Regarding mechanical factors, the presence of cantilevers has a negative effect as per the finite element analysis [4]. However, poor cleanability may induce peri-implantitis, resulting in marginal bone loss. Because the presence of a cantilever may impair cleanability, future studies that include not only marginal bone loss, but also cleanability as an outcome are required.

It is clear from the finite element analysis that cantilever length affects stress distribution around the implants [29]. Therefore, it is necessary to consider the effect of cantilever length; however, none of the studies included in this SR investigated this effect of cantilever length. However, although not included in this SR, Palmer et al. reported it [30]. They reported the mechanical complications that occurred in 29 cases in which prostheses with a cantilever were attached to a single implant placed in the anterior region; the most common complication was screw loosening. Hence, the cantilever length did not significantly affect the mechanical complication and the cantilever length of the complication group and non-complication group were 7.6 (7.1–7.9) mm and 7.3 (7.0–8.0) mm, respectively. However, it is not possible to conclude from this study alone whether the cantilever length affects the prognosis of implant treatment, and further research is required.

Another factor that could be related to implant prognosis but could not be investigated in this SR was the direction of the cantilever. Of the nine studies included in this SR, one study included only mesial cantilevers [23], two included only distal cantilevers [15, 18], five included both mesial and distal cantilevers [16, 17, 19, 21, 22], and this was unknown in another study [20]. None of these studies compared the results of implant treatments for the mesial and distal cantilevers. In view of the above, it would be difficult to conclude the relationship between the direction of the cantilever and prognosis. The number of supported implants is another important factor that may influence prognosis. However, no study has investigated this relationship, and it remains unclear. Therefore, it is necessary to accumulate further research results.

Two previously published SRs of cantilevered implant treatment included four studies each, two of which were shared between the two for a total of six included studies [5, 6], three of which were ineligible for this SR due to issues with study design. On the other hand, this SR was able to include six new studies that were not included in previous SRs, four of which were published after 2020 [17, 19, 22, 23]. Comparing the results of this study with those of a previous SRs, there was agreement that the presence of a cantilever had no effect on implant survival rate or marginal bone loss, although cantilevers increased the incidence of mechanical complications. As the number of studies included in this study was significantly greater than those in previous SRs and the results were consistent, the results of this study were able to provide stronger evidence. However, only one study was included regarding the occurrence of mechanical complications that was not included in previous SRs [22], indicating that there is still a lack of research on this outcome. As implant and screw materials have improved [31], and the advent of CAD/CAM, which allows for the creation of precise prosthetic devices, is rapidly increasing [32], further research is needed, especially regarding mechanical complications as an outcome. Recently, a SR has been published that focused on implant-supported single-unit crowns with cantilever extension in posterior regions [33]. Compared to this SR, whose included studies and outcomes were somewhat different, the results were generally consistent, such as the implant survival rate not being affected by the cantilever and an increase in mechanical complications, which suggests the possibility that the prognoses of single implant-supported and multiple implant-supported cantilever prostheses may be comparable.

There are several limitations in this study. Although we attempted to perform a meta-analysis by site regarding the occurrence of mechanical complications, this was impossible because there was an insufficient number of studies. On the other hand, although the relationship between bruxism and implant failure was known [34], only one study stated that it excluded patients with bruxism [18], another study stated that it included patients with bruxism [22], and the other studies did not state whether patients with bruxism were included or not. Therefore, it is unclear whether many of the included studies included patients with bruxism, and it is important to note that it is unclear whether the results of this SR can be applied to patients with bruxism. Furthermore, the number of studies included in this SR was not large, therefore further research is required to confirm these results.

## Conclusions

In response to the CQ, “Are cantilevered implant-supported fixed prostheses in dental implant treatment effective in cases of partial edentulism compared to conventional fixed prostheses?” it can be concluded that the possibility of mechanical complications increased. However, the possibility of affecting the survival rate of implants, patient satisfaction, and bone loss around the implant body was rare. Cantilevered implant-supported fixed prostheses can be applied without bone grafting procedures in cases of insufficient bone volume or anatomical constraints, which can be beneficial for patients.

Furthermore, there is a lack of clinical evidence regarding the effects of the number of implants, cantilever length, and differences between mesial and distal cantilevers; therefore, further studies are required.

## Data Availability

The datasets used and/or analyzed during the current study are available from the corresponding author upon reasonable request.

## Abbreviations

CQ: Clinical question
CENTRAL: Cochrane Central Register of Controlled Trials
PICO: Participant/patient, Intervention, comparison, and outcome
RCTs: Randomized controlled trials
SR: Systematic review
